# OPIOID DEPRESCRIBING: QUALITATIVE INSIGHTS INTO REDUCING MOTIVATION FOR UNHEALTHY SUBSTANCE USE

**DOI:** 10.1093/geroni/igae098.4358

**Published:** 2024-12-31

**Authors:** Lisa LaRowe, Sarah Stone, Heily Chavez Granados, Christine Ritchie

**Affiliations:** Massachusetts General Hospital, Boston, Massachusetts, United States; Massachusetts General Hospital, Boston, Massachusetts, United States; Massachusetts General Hospital, Boston, Massachusetts, United States; Mass General Hospital and Harvard Medical School, Boston, Massachusetts, United States

## Abstract

Nearly 38% of older adults in the United States have chronic pain, and opioids continue to be used frequently for chronic pain management in this population. While recommendations suggest limiting opioids in older adults, given limited effectiveness and associated harms, one potential consequence of deprescribing opioids is that patients may turn to non-prescribed opioids or nonopioid (e.g., alcohol, cannabis, nicotine) substances to self-medicate pain. We conducted 3 focus groups of older adults with chronic pain who were prescribed opioids (N = 10, 65-92 years old) to explore perspectives regarding factors that may influence motivation to use substances during opioid deprescribing. We also aimed to elicit preferences for an opioid deprescribing intervention that reduces risk for adverse substance use-related outcomes. Qualitative data were analyzed using rapid data analysis. Participants identified the following factors that may increase motivation to use substances during opioid deprescribing: intolerable pain, lack of access to alternative pain treatments, psychological distress, and history of substance use/abuse. Factors that may decrease motivation to use substances include: having access to alternative pain treatments, having access to psychosocial treatment, having adequate social support, and being aware of the risks/consequences of substance use. To mitigate risk that patients will use non-prescribed opioids and/or nonopioid substances to cope with pain during opioid deprescribing, participants highlighted the importance of having a clear plan for deprescribing, ensuring access to effective alternatives to opioids, utilizing psychosocial and other nonpharmacologic pain treatment strategies, and providing psychoeducation about the risks of substance use.

